# Supplementary material for: Trends in incidence, prevalence, and survival of breast cancer in the United Kingdom from 2000 to 2021
Source: Sci Rep. 2024 Aug 17;14:19069. doi: 10.1038/s41598-024-69006-1 (PMC11330450; doi:10.1038/s41598-024-69006-1)
Supplement: Supplementary file 1 — Supplementary Information. [file 41598_2024_69006_MOESM1_ESM.pdf]

Supplementary Information

Supplemental material – Trends in incidence, prevalence, and survival of breast cancer in the United Kingdom from 2000-2021

Nicola L Barclay, Edward Burn, Antonella Delmestri, Talita Duarte-Salles, Asieh Golozar, Wai Yi Man, Eng Hooi Tan, Ilona Tietzova, OPTIMA Consortium, Daniel Prieto Alhambra, Danielle Newby

Contents for Supplementary Information

S1: Clinical codelists for breast cancer 2

S2: Population attrition showing eligible patients for study from each database. 7

S3: Baseline characteristics of breast cancer patients at the time of diagnosis for CPRD Aurum. 8

S4: Annualised incidence rates for males stratified by database and age group. 9

S5: Prevalence in 2019 for breast cancer stratified by database, sex and age group. 10

S6: Annual percent change in prevalence of breast cancer across time for females in CPRD GOLD 11

S7: Annual percent change in prevalence of breast cancer across time for females in CPRD AURUM 12

S8: Annual percent change in prevalence of breast cancer across time for males in CPRD GOLD 13

S9: Annual percent change in prevalence of breast cancer across time for males in CPRD AURUM 14

S10: Number of patients at risk, died and censored across the follow-up period, stratified by sex and database. 15

S11: Survival (%) after one, five, and ten years after breast cancer diagnosis stratified by sex and database. 16

S12: Survival (%) after one and five years after breast cancer diagnosis stratified by calendar time of diagnosis, age and sex. 17

S1: Clinical codelists for breast cancer

The clinical codelists used for breast cancer are listed in the table below with the corresponding SNOMED concept ID OMOP concept ID and concept description. Only diagnosis records alone were used to identify cancer outcomes for this study. Different codelists were created for incident and prevalent definitions of breast cancer. We developed concept definitions using ATLAS, the OHDSI open-source platform (https://github.com/OHDSI/atlas). Clinical adjudicators reviewed the cohort definitions and associated concept sets.

Concept Id	Concept SNOMED Code	Concept Description
4157332	372064008	Malignant neoplasm of female breast
4112853	254837009	Malignant tumor of breast
4091465	188154003	Malignant neoplasm of upper-outer quadrant of female breast
4157447	372095001	Malignant neoplasm of male breast
4091464	188147009	Malignant neoplasm of nipple and areola of female breast
4092511	188151006	Malignant neoplasm of central part of female breast
4092512	188152004	Malignant neoplasm of upper-inner quadrant of female breast
442127	93924008	Primary malignant neoplasm of nipple of female breast
4091466	188155002	Malignant neoplasm of lower-outer quadrant of female breast
4095740	188153009	Malignant neoplasm of lower-inner quadrant of female breast
4091467	188156001	Malignant neoplasm of axillary tail of female breast
433148	93680004	Primary malignant neoplasm of areola of female breast
4092513	188157005	Malignant neoplasm overlapping lesion of breast
442126	93925009	Primary malignant neoplasm of nipple of male breast
436050	93681000	Primary malignant neoplasm of areola of male breast
4091469	188163001	Malignant neoplasm of nipple and areola of male breast
4091471	188168005	Malignant neoplasm of ectopic site of male breast
4095741	188159008	Malignant neoplasm of ectopic site of female breast
133711	109886000	Overlapping malignant neoplasm of female breast
135489	93884005	Primary malignant neoplasm of male breast
137809	93796005	Primary malignant neoplasm of female breast

432263	93874009	Primary malignant neoplasm of lower inner quadrant of female breast
432845	93745008	Primary malignant neoplasm of central portion of female breast
436353	94117003	Primary malignant neoplasm of upper outer quadrant of female breast
440956	94115006	Primary malignant neoplasm of upper inner quadrant of female breast
441513	372092003	Primary malignant neoplasm of axillary tail of breast
441515	93876006	Primary malignant neoplasm of lower outer quadrant of female breast
759932	1080101000119100	Infiltrating duct carcinoma of left female breast
759933	1080181000119100	Infiltrating duct carcinoma of right female breast
761170	1563576100011910 0	Infiltrating duct carcinoma of bilateral female breasts
765123	353511000119101	Primary malignant neoplasm of female right breast
4003684	109887009	Overlapping malignant neoplasm of male breast
4080865	278054005	Infiltrating lobular carcinoma of breast
4110861	254843006	Familial cancer of breast
4112073	254839007	Scirrhous carcinoma of breast
4112074	254840009	Inflammatory carcinoma of breast
4112854	254844000	Malignant phyllodes tumor of breast
4113637	286894008	Carcinoma of breast - lower inner quadrant
4113638	286897001	Carcinoma of breast - axillary tail
4116071	254838004	Carcinoma of breast
4117850	286893002	Carcinoma of breast - upper inner quadrant
4117851	286895009	Carcinoma of breast - upper outer quadrant
4117852	286896005	Carcinoma breast - lower outer quadrant
4142116	427685000	HER2-positive carcinoma of breast
4155292	372094002	Malignant neoplasm of axillary tail of breast
4157448	372096000	Carcinoma of male breast
4158563	373089009	Primary malignant neoplasm of breast upper inner quadrant

4160780	373083005	Malignant neoplasm of breast upper outer quadrant
4162253	372137005	Primary malignant neoplasm of breast
4187848	373081007	Malignant neoplasm of breast lower outer quadrant
4187849	373082000	Malignant neoplasm of breast upper inner quadrant
4187850	373088001	Primary malignant neoplasm of breast upper outer quadrant
4187851	373090000	Primary malignant neoplasm of breast lower inner quadrant
4188544	373080008	Malignant neoplasm of breast lower inner quadrant
4188545	373091001	Primary malignant neoplasm of breast lower outer quadrant
4216891	417181009	Hormone receptor positive malignant neoplasm of breast
4237178	408643008	Infiltrating duct carcinoma of breast
4246036	93776002	Primary malignant neoplasm of ectopic female breast tissue
4246810	93777006	Primary malignant neoplasm of ectopic male breast tissue
4330242	431396003	Human epidermal growth factor 2 negative carcinoma of breast
35622134	763479005	Metaplastic carcinoma of breast
35624616	767444009	Germline BRCA-mutated HER2-negative metastatic breast cancer
36684817	353421000119109	Primary malignant neoplasm of axillary tail of left female breast
36684818	353431000119107	Primary malignant neoplasm of female left breast
36684819	353441000119103	Primary malignant neoplasm of central portion of female left breast
36684820	353501000119104	Primary malignant neoplasm of axillary tail of right female breast
36684821	353521000119108	Primary malignant neoplasm of central portion of female right breast
36684948	459391000124109	Metastatic human epidermal growth factor 2 positive carcinoma of breast
36684950	459411000124109	Metastatic collecting duct carcinoma
36712719	1080151000119100	Infiltrating ductal carcinoma of upper inner quadrant of left female breast
36712720	1080161000119100	Infiltrating ductal carcinoma of upper outer quadrant of left female breast
36712721	1080191000119100	Infiltrating ductal carcinoma of central portion of right female breast
36712722	1080231000119100	Infiltrating ductal carcinoma of upper inner quadrant of right female breast

36712723	1080241000119100	Infiltrating ductal carcinoma of upper outer quadrant of right female breast
36712724	1080261000119100	Infiltrating lobular carcinoma of left female breast
36712725	1080341000119100	Infiltrating lobular carcinoma of right female breast
36712934	1563580100011910 0	Primary malignant neoplasm of bilateral female breasts
36716497	722524005	Primary invasive pleomorphic lobular carcinoma of breast
36717260	1080111000119100	Infiltrating ductal carcinoma of central portion of left female breast
36717587	722832009	Primary solid papillary carcinoma with invasion of breast
37016439	45221000119105	Primary invasive malignant neoplasm of female breast
37017351	713609000	Invasive carcinoma of breast
37018660	96291000119105	Primary malignant inflammatory neoplasm of female breast
37208047	354491000119109	Primary malignant neoplasm of left male breast
37208048	354591000119108	Primary malignant neoplasm of right male breast
37208322	1080091000119100	Infiltrating ductal carcinoma of axillary tail of left female breast
37208324	1080121000119100	Infiltrating ductal carcinoma of lower inner quadrant of left female breast
37208325	1080131000119100	Infiltrating ductal carcinoma of lower outer quadrant of left female breast
37208326	1080171000119100	Infiltrating ductal carcinoma of axillary tail of right female breast
37208328	1080201000119100	Infiltrating ductal carcinoma of lower inner quadrant of right female breast
37208329	1080211000119100	Infiltrating ductal carcinoma of lower outer quadrant of right female breast
37310457	1082701000112100	Locally advanced breast cancer
40480215	444604002	Mixed ductal and lobular carcinoma of breast
40480651	444712000	Mucinous carcinoma of breast
40486563	447782002	Carcinoma of female breast
40492507	448952004	Infiltrating duct carcinoma of female breast
45768522	706970001	Triple-negative breast cancer
46270923	708921005	Carcinoma of central portion of breast
44811954	94361000000105	Breast cancer detected by national screening programme*

36712738	1081551000119100	Recurrent primary malignant neoplasm of left female breast*
36712739	1081561000119100	Recurrent primary malignant neoplasm of right female breast*
4198446	315004001	Metastasis from malignant tumor of breast*
4201477	314955001	Local recurrence of malignant tumor of breast*

Note. All codes used for estimates of incidence and prevalence, except for those indicated with * for which the codes were used to estimate prevalence only.

S2: Population attrition showing eligible patients for study from each database.

		Females		Males	
**Database**	**Reason**	**N**	**N excluded**	**N**	**N excluded**
GOLD	Starting population	17054819		17054819	
	Missing year of birth	17054819	0	17054819	0
	Missing sex	17054819	0	17054819	0
	Cannot satisfy age criteria during the study period based on year of birth	15210165	1844654	15210165	1844654
	No observation time available during study period	13978229	1231936	13978229	1231936
	Doesn't satisfy age criteria during the study period	13978229	0	13978229	0
	Prior history requirement not fulfilled during study period	12254874	1723355	12254874	1723355
	Not Female	6275193	5979681	-	-
	Not Male	-	-	5979681	6275193
	No observation time available after applying age and prior history criteria	5848436	426757	5539681	440000
	Starting analysis population	5848436		5539681	
	Estimating prevalence	5848436		5539681	
	Excluded due-prior event (do not pass outcome washout during study period)	5832192	16244	5539598	83
	Estimating incidence	5832192	0	5539598	0
	With a cancer diagnosis	85400	5746792	507	5539091
	Cancer diagnosis not on same date as death		416		2
	Estimating survival	84984		505	
Aurum	Starting population	39999011		39999011	
	Missing year of birth	39999011	0	39999011	0
	Missing sex	39999011	0	39999011	0
	Cannot satisfy age criteria during the study period based on year of birth	34833388	5165623	34833388	5165623
	No observation time available during study period	29190480	5642908	29190480	5642908
	Doesn't satisfy age criteria during the study period	29190480	0	29190480	0
	Prior history requirement not fulfilled during study period	25483313	3707167	25483313	3707167
	Not Female	13057840	12425473	-	-
	Not Male	-	-	12425473	13057840
	No observation time available after applying age and prior history criteria	12496239	561601	11844621	580852
	Starting analysis population	12496239		11844621	
	Estimating prevalence	12496239		11844621	
	Excluded due-prior event (do not pass outcome washout during study period)	12444037	52202	11844346	275
	Estimating incidence	12444037	0	11844346	0
	With a cancer diagnosis	156807	12287230	1019	11843327
	Cancer diagnosis not on same date as death		329		2
	Estimating survival	156478		1017	

S3: Baseline characteristics of breast cancer patients at the time of diagnosis for CPRD Aurum.

**Database**	**CPRD Aurum**
**Number of patients**	157826
**Sex: Male (N[%])**	1019 (0.6%)
**Age (Median [IQR])**	63 (52-73)
**Age Groups years N (%)**	
18-29	492 (0.30%)
30-39	5907 (3.70%)
40-49	22134 (14.0%)
50-59	38276 (24.3%)
60-69	38953 (24.7%)
70-79	28350 (18.0%)
80-89	18877 (12.0%)
90+	4837 (3.1%)
**Prior history days**	
median [IQR]	5639 (2669-9528)
**General conditions (any time prior)**	
Atrial fibrillation	5971 (3.8%)
Cerebrovascular disease	5748 (3.6%)
Chronic liver disease	429 (0.3%)
Chronic obstructive lung disease	5421 (3.4%)
Coronary arteriosclerosis	564 (0.4%)
Dementia	2138 (1.4%)
Depressive disorder	24842 (15.7%)
Diabetes mellitus	13271 (8.4%)
Gastroesophageal reflux disease	4311 (2.7%)
Gastrointestinal haemorrhage	8472 (5.4%)
Heart failure	2931 (1.9%)
Hyperlipidemia	12292 (7.8%)
Hypertensive disorder	46891 (29.7%)
Ischemic heart disease	8149 (5.2%)
Osteoarthritis	34762 (22.0%)
Peripheral vascular disease	1340 (0.8%)
Pulmonary embolism	1397 (0.9%)
Renal impairment	12714 (8.1%)
Venous thrombosis	6944 (4.4%)

IQR: interquartile range

S4: Annualised incidence rates for males stratified by database and age group.

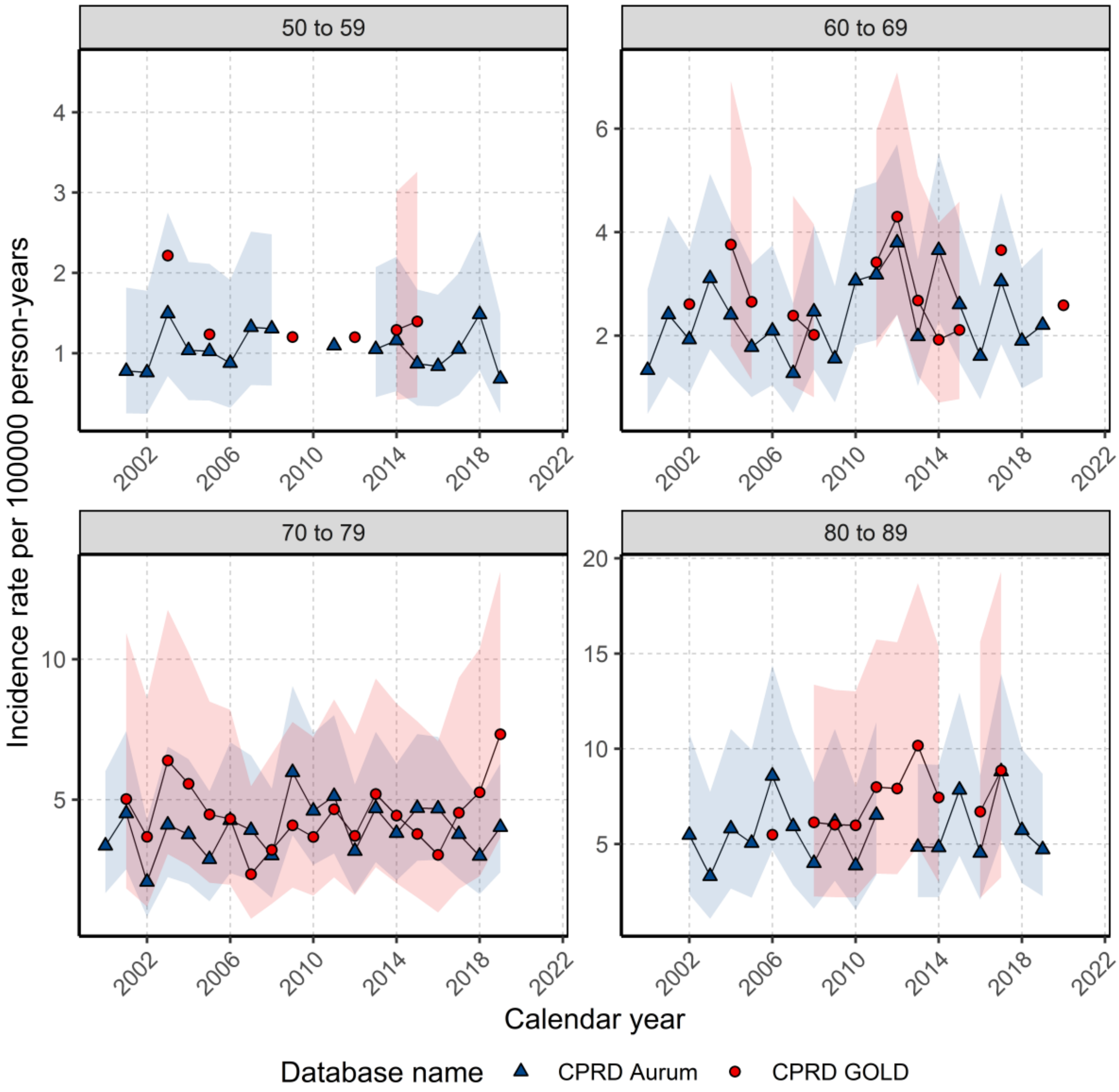

S5: Prevalence in 2019 for breast cancer stratified by database, sex and age group.

Sex	Database	Age Group (years)	n cases	n population	Prevalence (95%CI)
Female	CPRD GOLD	18-29	32	280123	0.011% (0.008% - 0.016%)
		30-39	400	273050	0.146% (0.133% - 0.162%)
		40-49	2064	257352	0.802% (0.768% - 0.837%)
		50-59	6747	277582	2.431% (2.374% - 2.489%)
		60-69	8701	220684	3.943% (3.862% - 4.025%)
		70-79	9311	176503	5.275% (5.172% - 5.381%)
		80-89	4391	96338	4.558% (4.428% - 4.691%)
		90 +	1040	29079	3.576% (3.369% - 3.796%)
	CPRD Aurum	18-29	107	1080945	0.010% (0.008% - 0.012%)
		30-39	1634	991323	0.165% (0.157% - 0.173%)
		40-49	7443	871074	0.854% (0.835% - 0.874%)
		50-59	23150	888716	2.605% (2.572% - 2.638%)
		60-69	28469	671981	4.237% (4.189% - 4.285%)
		70-79	32515	549649	5.916% (5.854% - 5.978%)
		80-89	17486	307525	5.686% (5.605% - 5.768%)
		90 +	4410	91182	4.836% (4.699% - 4.978%)
Male	CPRD GOLD	18-29	-	-	-
		30-39	-	-	-
		40-49	6	267294	0.002% (0.001% - 0.005%)
		50-59	22	284110	0.008% (0.005% - 0.012%)
		60-69	33	217106	0.015% (0.011% - 0.021%)
		70-79	56	160256	0.035% (0.027% - 0.045%)
		80-89	30	71121	0.042% (0.030% - 0.060%)
		90 +	6	13427	0.045% (0.020% - 0.097%)
	CPRD Aurum	18-29	-	-	-
		30-39	6	1023263	0.001% (0.000% - 0.001%)
		40-49	29	933124	0.003% (0.002% - 0.004%)
		50-59	68	931059	0.007% (0.006% - 0.009%)
		60-69	118	672155	0.018% (0.015% - 0.021%)
		70-79	208	500455	0.042% (0.036% - 0.048%)
		80-89	162	231431	0.070% (0.060% - 0.082%)
		90 +	41	44046	0.093% (0.069% - 0.126%)

S6: Annual percent change in prevalence of breast cancer across time for females in CPRD GOLD

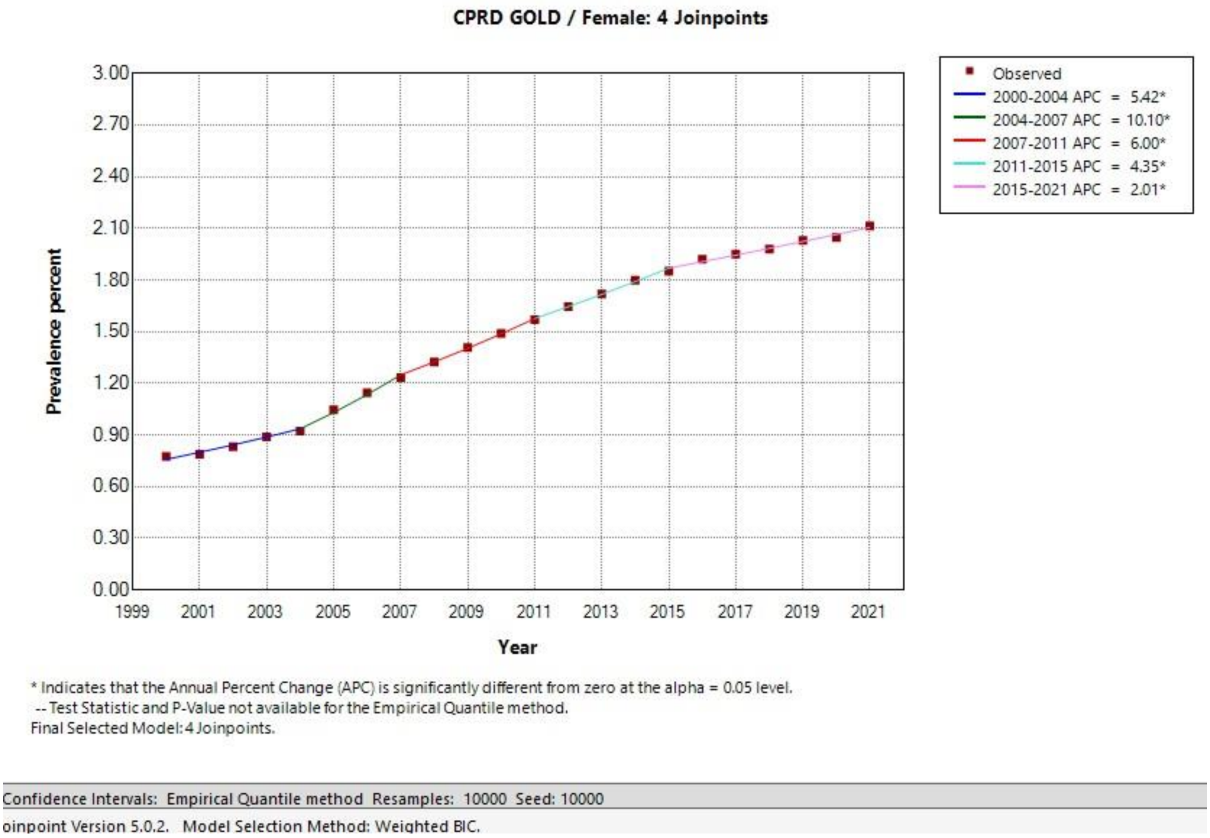

S7: Annual percent change in prevalence of breast cancer across time for females in CPRD AURUM

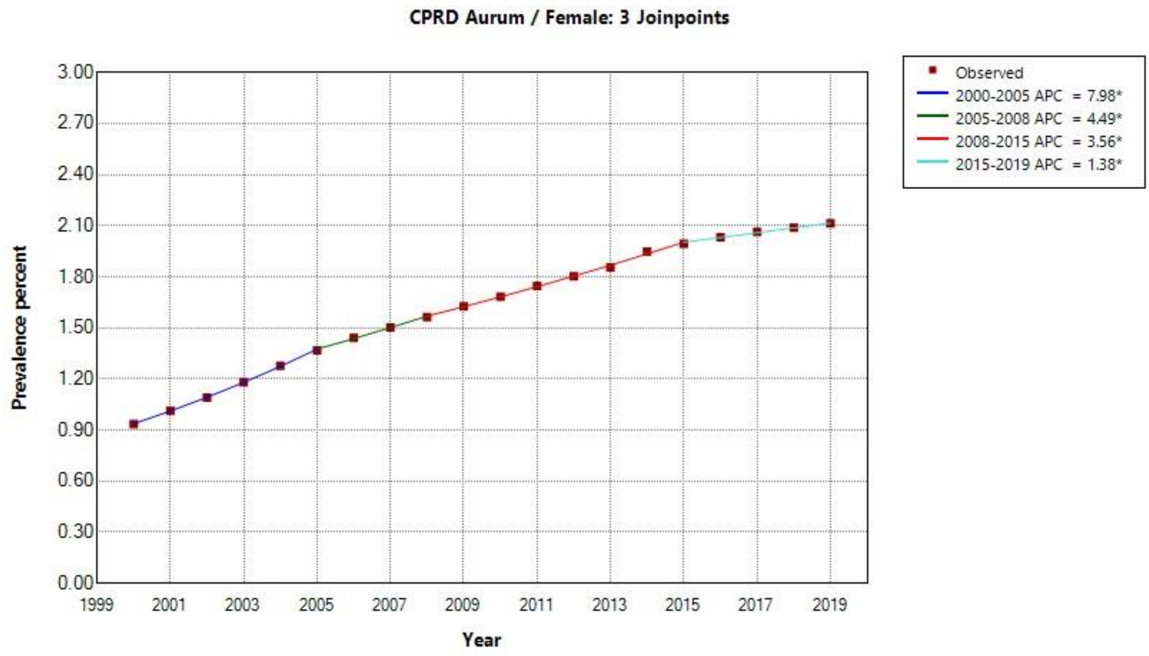

* Indicates that the Annual Percent Change (APC) is significantly different from zero at the alpha = 0.05 level.
-- Test Statistic and P-Value not available for the Empirical Quantile method.
Final Selected Model: 3 Joinpoints.

Confidence Intervals: Empirical Quantile method Resamples: 10000 Seed: 10000
Joinpoint Version 5.0.2. Model Selection Method: Weighted BIC.

S8: Annual percent change in prevalence of breast cancer across time for males in CPRD GOLD

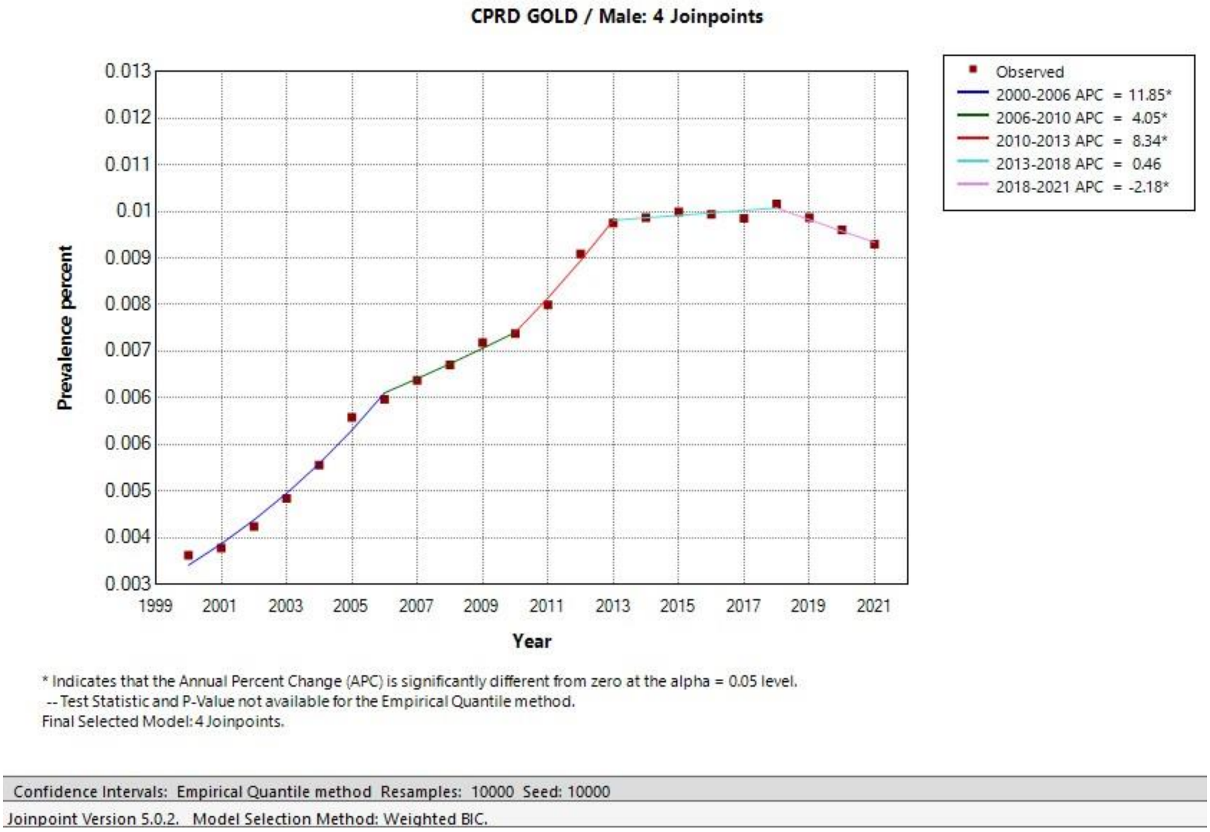

S9: Annual percent change in prevalence of breast cancer across time for males in CPRD AURUM

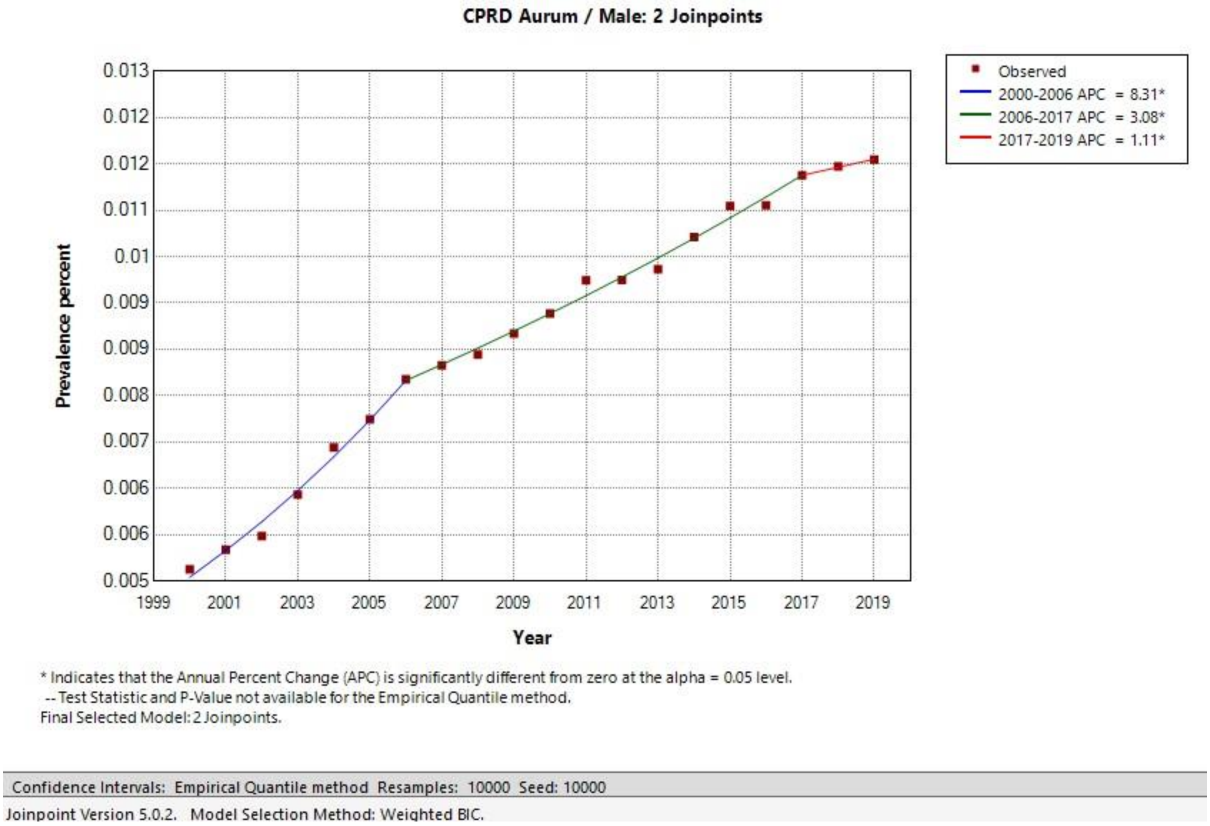

S10: Number of patients at risk, died and censored across the follow-up period, stratified by sex and database.

Sex	Database	Time (years after diagnosis)	Number at risk	Number Died	Number censored
Female	GOLD	0	84984	7239	13969
		2	63776	4715	11532
		4	47529	2959	9568
		6	35002	1915	8083
		8	25004	1278	6434
		10	17292	885	5544
		12	10863	511	3919
		14	6433	286	2622
		16	3525	140	2214
		18	1171	41	845
		20	285	5	276
Male		0	505	61	91
		2	353	47	68
		4	238	26	47
		6	165	17	37
		8	111	10	31
		10	70	11	22
		12	37	0	14
		14	23	0	11
		16	12	<5	6
		18	5	0	<5
Female	AURUM	0	156478	12605	25616
		2	118257	8583	20467
		4	89207	5576	16878
		6	66753	3814	13679
		8	49260	2592	11304
		10	35364	1883	9342
		12	24139	1255	7664
		14	15220	757	6324
		16	8139	346	4541
		18	3252	116	3136
Male		0	1017	105	162
		2	750	82	140
		4	528	64	101
		6	363	32	68
		8	263	30	58
		10	175	20	44
		12	111	7	31
		14	73	6	28
		16	39	0	21
		18	18	<5	13

S11: Survival (%) after one, five, and ten years after breast cancer diagnosis stratified by sex and database.

Sex	Database	Time (years after diagnosis)	% Survival (95% CI)
Male	GOLD	1	92.94 (90.67 - 95.26)
		5	69.03 (64.50 - 73.87)
		10	51.34 (45.55 - 57.87)
Female		1	95.14 (94.99 - 95.28)
		5	80.19 (79.88 - 80.49)
		10	68.44 (68.02 - 68.86)
Male	Aurum	1	93.14 (91.56 - 94.74)
		5	71.82 (68.69 - 75.09)
		10	52.77 (48.69 - 57.19)
Female		1	95.38 (95.27 - 95.49)
		5	80.81 (80.59 - 81.03)
		10	68.78 (68.48 - 69.08)

CI: confidence interval

S12: Survival (%) after one and five years after breast cancer diagnosis stratified by calendar time of diagnosis, age and sex.

Calendar Year of Diagnosis	Age Group (years)	Time (years after diagnosis)	% Survival (95% CI) for females	% Survival (95% CI) for males
2000-2004	18-29	1	97.73 (93.42 - 100.00)	NA (NA - NA)
		5	79.19 (67.20 - 93.31)	NA (NA - NA)
	30-39	1	95.08 (93.40 - 96.78)	NA (NA - NA)
		5	78.30 (74.99 - 81.75)	NA (NA - NA)
	40-49	1	97.86 (97.22 - 98.50)	NA (NA - NA)
		5	85.01 (83.40 - 86.65)	NA (NA - NA)
	50-59	1	97.35 (96.87 - 97.84)	100.00 (100.00 - 100.00)
		5	88.24 (87.25 - 89.25)	83.33 (64.70 - 100.00)
	60-69	1	95.59 (94.92 - 96.28)	95.83 (88.16 - 100.00)
		5	84.35 (83.13 - 85.59)	74.56 (58.87 - 94.43)
	70-79	1	91.60 (90.55 - 92.67)	100.00 (100.00 - 100.00)
		5	69.64 (67.86 - 71.48)	85.94 (74.06 - 99.72)
	80-89	1	84.32 (82.63 - 86.04)	87.50 (67.34 - 100.00)
		5	47.60 (45.17 - 50.16)	72.92 (46.80 - 100.00)
	90 +	1	73.46 (69.34 - 77.82)	NA (NA - NA)
		5	22.57 (18.47 - 27.58)	NA (NA - NA)
2005-2009	18-29	1	97.28 (93.62 - 100.00)	NA (NA - NA)
		5	87.59 (79.82 - 96.11)	NA (NA - NA)
	30-39	1	98.31 (97.44 - 99.19)	NA (NA - NA)
		5	85.54 (83.05 - 88.10)	NA (NA - NA)
	40-49	1	98.49 (98.07 - 98.91)	100.00 (100.00 - 100.00)
		5	87.64 (86.46 - 88.84)	90.00 (73.20 - 100.00)
	50-59	1	98.26 (97.94 - 98.58)	90.00 (77.77 - 100.00)
		5	90.59 (89.85 - 91.34)	73.23 (55.50 - 96.62)
	60-69	1	96.91 (96.50 - 97.32)	90.00 (79.88 - 100.00)
		5	86.62 (85.80 - 87.46)	71.43 (56.36 - 90.54)
	70-79	1	92.90 (92.14 - 93.68)	92.17 (84.04 - 100.00)
		5	72.99 (71.61 - 74.39)	54.06 (40.11 - 72.84)
	80-89	1	86.24 (84.95 - 87.55)	86.54 (73.46 - 100.00)
		5	49.62 (47.61 - 51.71)	44.56 (25.71 - 77.23)
	90 +	1	73.48 (70.11 - 77.01)	NA (NA - NA)
		5	21.20 (17.78 - 25.27)	NA (NA - NA)
2010-2014	18-29	1	98.78 (96.43 - 100.00)	NA (NA - NA)
		5	83.33 (74.72 - 92.95)	NA (NA - NA)
	30-39	1	97.82 (96.69 - 98.95)	NA (NA - NA)
		5	84.42 (81.24 - 87.72)	NA (NA - NA)
	40-49	1	98.73 (98.34 - 99.11)	100.00 (100.00 - 100.00)
		5	89.82 (88.66 - 91.00)	90.91 (75.41 - 100.00)

	50-59	1	98.34 (97.99 - 98.68)	100.00 (100.00 - 100.00)
		5	91.47 (90.63 - 92.31)	69.24 (55.25 - 86.78)
	60-69	1	98.01 (97.67 - 98.36)	93.33 (86.31 - 100.00)
		5	88.60 (87.73 - 89.49)	73.41 (59.27 - 90.92)
	70-79	1	93.99 (93.26 - 94.72)	80.84 (68.97 - 94.75)
		5	74.85 (73.35 - 76.38)	43.76 (27.72 - 69.10)
	80-89	1	87.59 (86.33 - 88.86)	NA (NA - NA)
		5	51.96 (49.76 - 54.27)	NA (NA - NA)
	90 +	1	73.77 (70.47 - 77.23)	NA (NA - NA)
		5	25.51 (21.54 - 30.22)	NA (NA - NA)
2015-2019	18-29	1	97.44 (92.60 - 100.00)	NA (NA - NA)
		5	85.38 (74.26 - 98.16)	NA (NA - NA)
	30-39	1	98.29 (97.12 - 99.47)	100.00 (100.00 - 100.00)
		5	89.04 (85.62 - 92.60)	NA (NA - NA)
	40-49	1	98.40 (97.83 - 98.97)	90.00 (73.20 - 100.00)
		5	89.01 (87.25 - 90.81)	78.75 (56.41 - 100.00)
	50-59	1	98.97 (98.65 - 99.29)	81.57 (66.76 - 99.66)
		5	92.41 (91.40 - 93.42)	81.57 (66.76 - 99.66)
	60-69	1	97.78 (97.32 - 98.25)	97.22 (92.00 - 100.00)
		5	89.17 (88.00 - 90.36)	62.64 (44.20 - 88.77)
	70-79	1	93.96 (93.09 - 94.84)	87.05 (71.80 - 100.00)
		5	75.90 (73.97 - 77.88)	50.78 (30.33 - 85.03)
	80-89	1	88.07 (86.53 - 89.65)	NA (NA - NA)
		5	56.19 (53.17 - 59.37)	NA (NA - NA)
	90 +	1	77.27 (73.23 - 81.54)	NA (NA - NA)
		5	24.31 (18.66 - 31.66)	NA (NA - NA)
2020-2021	18-29	1	100.00 (100.00 - 100.00)	NA (NA - NA)
	30-39	1	97.06 (93.81 - 100.00)	NA (NA - NA)
	40-49	1	98.28 (96.90 - 99.69)	NA (NA - NA)
	50-59	1	98.40 (97.50 - 99.30)	NA (NA - NA)
	60-69	1	97.34 (96.16 - 98.54)	NA (NA - NA)
	70-79	1	93.33 (91.24 - 95.47)	NA (NA - NA)
	80-89	1	87.97 (84.56 - 91.52)	NA (NA - NA)
	90 +	1	79.14 (70.83 - 88.43)	NA (NA - NA)

CI: confidence interval
